# The acute effects of dynamic stretching on the neuromuscular system are independent of the velocity

**DOI:** 10.1113/EP092217

**Published:** 2025-01-06

**Authors:** Denis César Leite Vieira, Nicolas Babault, Marion Hitier, João Luiz Quagliotti Durigan, Martim Bottaro

**Affiliations:** ^1^ INSERM UMR1093‐CAPS & Centre d'Expertise de la Performance UFR des Sciences du Sport, Université de Bourgogne Dijon France; ^2^ Strength and Conditioning Research Laboratory, College of Physical Education University of Brasília Brasília Brazil; ^3^ Graduate Program of Physical Education, Department of Physical Education Catholic University of Brasilia Taguatinga Brazil; ^4^ Laboratory of Muscle and Tendon Plasticity, Graduate Program of Rehabilitation Sciences University of Brasilia Brasília Brazil

**Keywords:** muscle performance, neural adaptations, peripheral adaptations, stretch, warm‐up

## Abstract

This study examined the acute effects of dynamic stretching at different velocities on the neuromuscular system. Fourteen participants underwent four experimental sessions in random order: (1) control (lying at rest with the ankle in a neutral position); (2) slow velocity dynamic stretching (50 beats/min; SLOW_DS_); (3) moderate velocity dynamic stretching (70 beats/min; MOD_DS_); and (4) fast velocity dynamic stretching (90 beats/min; FAST_DS_). The stretching protocols consisted of four sets of 10 repetitions and targeted the plantar flexor muscles of the right ankle. Assessments included corticospinal excitability (via motor‐evoked potential—MEP/*M*_max_), spinal reflex activity (via H‐reflex—*H*_max_/*M*_max_), muscle contractile properties (peak twitch torque; PTT), maximal voluntary contraction (MVC), and maximal range of motion (ROM_max_). Dynamic stretching did not affect MEP/*M*_max_ and MVC of the plantar flexor muscles (*P* > 0.05). All stretching protocols similarly reduced soleus *H*_max_/*M*_max_ (*P* < 0.05), and increased PTT (*P* < 0.05). Additionally, all conditions, including control, similarly increase ROM_max_ (*P* < 0.05, and Cohen's *d* value of −0.39, −0.28, −0.38 and −0.29 for CON, SLOW_DS_, MOD_DS_ and FAST_DS_, respectively). Therefore, dynamic stretching reduces spinal reflex activity and enhances muscle contractile properties irrespective of movement velocity without impairing corticospinal excitability and MVC.

## Abstract (highlights)

- **What is the central question of this study?**
  What are the acute effects of dynamic stretching at different velocities on the neuromuscular system?
- **What is the main finding and its importance?**
  Spinal reflex activity decreased immediately after dynamic stretching, regardless of velocity. Nonetheless, spinal reflex activity returned to baseline values 7 min after dynamic stretching. Therefore, this effects on spinal reflex are transient. Evoked contractile properties increased immediately after dynamic stretching, regardless of velocity, and remained higher than baseline values 7 min after. Therefore, dynamic streching may be useful to promote peripherical adaptation that could have an impact on performance.

## 1 INTRODUCTION

Static stretching is the most popular form of stretching practiced by physically active individuals to increase joint range of motion (Babault et al., 2021). Despite its positive effects for enhancing joint range of motion, most literature reports that prolonged static stretching in isolation (i.e. greater than 60 s per muscle group, and without a dynamic warm‐up) negatively impacts muscle strength and power (Behm & Chaouachi, 2011; Behm et al., 2016). Thus, dynamic stretching has been recommended as an alternative, since it can improve joint range of motion without impairing or even improving muscle performance (Babault et al., 2021; Opplert & Babault, 2018). Indeed, dynamic stretching has been shown to enhance jump performance, and maximal force production (Opplert & Babault, 2018; Pamboris et al., 2018, 2019). Consequently, practitioners widely consider dynamic stretching as the best form of stretching before exercise training (Babault et al., 2021).

The dynamic stretching studies reporting performance improvements might be explained by multiple adaptations within the neuromuscular system, including both peripheral (i.e. muscle–tendon unit) and neural mechanisms (Opplert & Babault, 2018, 2019; Vieira et al., 2021). For instance, it has been suggested that dynamic stretching increases local temperature and may promote peripheral adaptations such as decreased muscle viscoelasticity and displacement of the tendon unit (Mcnair et al., 2001; Pamboris et al., 2018; Samukawa et al., 2011). Consequently, this leads to decreased muscle–tendon stiffness and improved joint range of motion (Samukawa et al., 2011; Vieira et al., 2021). Other studies reported that dynamic stretching impacts neural efferent drive to the muscle (Fletcher, 2010; Vieira et al., 2021). Indeed, dynamic stretching changes corticospinal excitability and spinal motor neuron activity (Clark et al., 2014; Opplert et al., 2020). Therefore, this affects the ability of the muscle to produce force (Opplert & Babault, 2019; Opplert et al., 2020; Trajano et al., 2017; Vieira et al., 2021).

However, dynamic stretching protocols may vary according to the volume, intensity and velocity (Fletcher, 2010; Mizuno, 2017; Opplert & Babault, 2018; Pamboris et al., 2019; Takeuchi et al., 2022; Turki et al., 2012). The changes in the neuromuscular system during and immediately after stretching movements performed at different velocities have been explored (Fletcher, 2010; Mizuno, 2022; Opplert & Babault, 2018; Pamboris et al., 2018; Takeuchi et al., 2022). For instance, previous studies revealed greater cortical activity at faster movements during muscle lengthening and shortening (Lucier et al., 1975; Saito et al., 2018). Conversely, an inhibitory effect on spinal reflex activity was found during the muscle lengthening at faster than slower movement velocities (Budini & Tilp, 2016; Duclay et al., 2009). Nonetheless, these studies reported above did not use dynamic stretching protocols commonly used during warm‐up routines, that is, they investigated dynamic stretching protocols with passive movements, low volume or movement velocity controlled by angular velocity using sophisticated and non‐ecologically applied devices (Duclay et al., 2009; Guissard et al., 2001; Lucier et al., 1975; Saito et al., 2018).

An ecological form, traditionally, applied in studies to control dynamic stretching velocity, is the movement frequency from a metronome (Fletcher, 2010; Mizuno, 2022; Opplert et al., 2020; Pamboris et al., 2018, 2019). Although some studies with ecologically applied dynamic stretching protocols verified the acute effect of dynamic stretching velocity on the neuromuscular system, divergent results are regularly reported (Fletcher, 2010; Mizuno, 2022; Pamboris et al., 2019). For instance, Pamboris et al. (2019) found a greater increase in muscle stiffness and eccentric torque after dynamic stretching performed at a frequency of 50 beats/min compared to 100 beats/min. Conversely, Fletcher (2010) reported a greater improvement in jump performance after dynamic stretching performed at a frequency of 100 beats/min compared to 50 beats/min. It was suggested, from the electromyogram (EMG) amplitude recorded during the jump, that the greater improvement in jump performance following the dynamic stretching at a higher frequency resulted from neural mechanisms (Fletcher, 2010).

Nonetheless, EMG amplitude, a measure of muscle excitation (Vigotsky et al., 2017), is not sensitive to differentiating adaptations in specific sites within the neuromuscular system, as EMG amplitude may be influenced by some central parameters, for instance cortical and spinal adaptations, as well as peripherical changes, such as sarcolemma membrane excitability (Trajano & Blazevich, 2021; Trajano et al., 2017; Vigotsky et al., 2017). Therefore, the acute effects of dynamic stretching at different velocities on specific sites within the neuromuscular system, such as corticospinal excitability, spinal reflex activity and muscle contractile properties, remain unclear. Additionally, dynamic stretching is generally conducted as a warm‐up component for subsequent training or competition (Babault et al., 2021, 2022, 2023; Pamboris et al., 2019), and therefore, the effects are expected to last for several minutes. However, studies exploring the time course effects of dynamic stretching velocity on the neuromuscular system are scarce (Pamboris et al., 2018, 2019).

Therefore, using an ecological dynamic stretching protocol with velocity controlled by movement frequency, this study aimed to verify the effects of dynamic stretching velocity on muscle mechanism (strength, stiffness, maximal range of motion (ROM_max_)), and various sites of the neuromuscular system while considering corticospinal excitability and spinal reflex activity. Additionally, the second aim of this study was to verify the time course effect of these neuromuscular acute effects from dynamic stretching at various velocities. Studies using non‐ecologically applied dynamic stretching protocols indicated that corticospinal excitability was higher and spinal activity was reduced in faster than slower dynamic stretching velocities (Budini & Tilp, 2016; Duclay et al., 2009; Guissard et al., 2001; Lucier et al., 1975; Saito et al., 2018). Thus, we hypothesized that dynamic stretching at faster velocities might increase corticospinal activity and reduce spinal excitability compared with slower velocities.

## 2 METHODS

### 2.1 Ethical approval

The study received approval from the Ethics Committee for Research in STAPS (CERSTAPS IRB00012476‐2023‐17‐02‐229). All individuals were fully informed about the experimental procedure and purpose of the study, and they read and signed an informed consent form. The study followed the standards set out by the *Declaration of Helsinki*, except for the clause 35, that is, registration in a public database.

### 2.2 Participants

Fourteen volunteers (11 men and 3 women) participated in this study. The mean ± standard deviation (SD) age, height, body mass and physical activity per week for men and women were, respectively, 25.4 ± 3.6 and 32.3 ± 3.8 years, 169.6 ± 9.28 and 162.7 ± 7.8 cm, 77.6 ± 14.6 and 69.3 ± 6.0 kg, and 4.5 ± 1.6 and 4.0 ± 1.0 h. None of the participants reported any lower limb injuries or back pain in the last 3 months nor any specific hamstrings or triceps surae injuries in the last 2 years. Before inclusion, participants answered the transcranial magnetic stimulation (TMS) readiness questionnaire and were considered able to do TMS procedures (Rossi et al., 2011). During the present experiment, participants were asked to maintain their regular physical activities and dietary intake. They were also advised to refrain from intensive activity for at least two days before an experimental session. The sample size was calculated a priori using G*Power (version 3.1.9.6) based on previous studies that aimed to verify the acute effect of stretching exercises on MEP/*M*_max_, which reported a partial etta square (ƞ_p_^2^) ranging from 0.18 to 0.23, an effect size between 0.47 and 0.55, a power of 0.8, and a probability error 0.05 (Opplert et al., 2020; Pulverenti et al., 2019). Thus, a sample of 13–14 volunteers was deemed sufficient.

### 2.3 Experimental procedures

A randomized, controlled and crossover design was used to explore acute neuromuscular adaptations from dynamic stretching performed at different movement velocities. Participants visited our lab on five occasions separated by 72 h. The first visit was used to familiarize volunteers to all test procedures and dynamic stretching protocols. The four other experimental sessions (randomly presented) were: (1) control session; (2) dynamic stretching at slow velocity (50 beats/min; SLOW_DS_); (3) dynamic stretching at moderate velocity (70 beats/min; MOD_DS_); and (4) dynamic stretching at fast velocity (90 beats/min; FAST_DS_).

**FIGURE 1:**
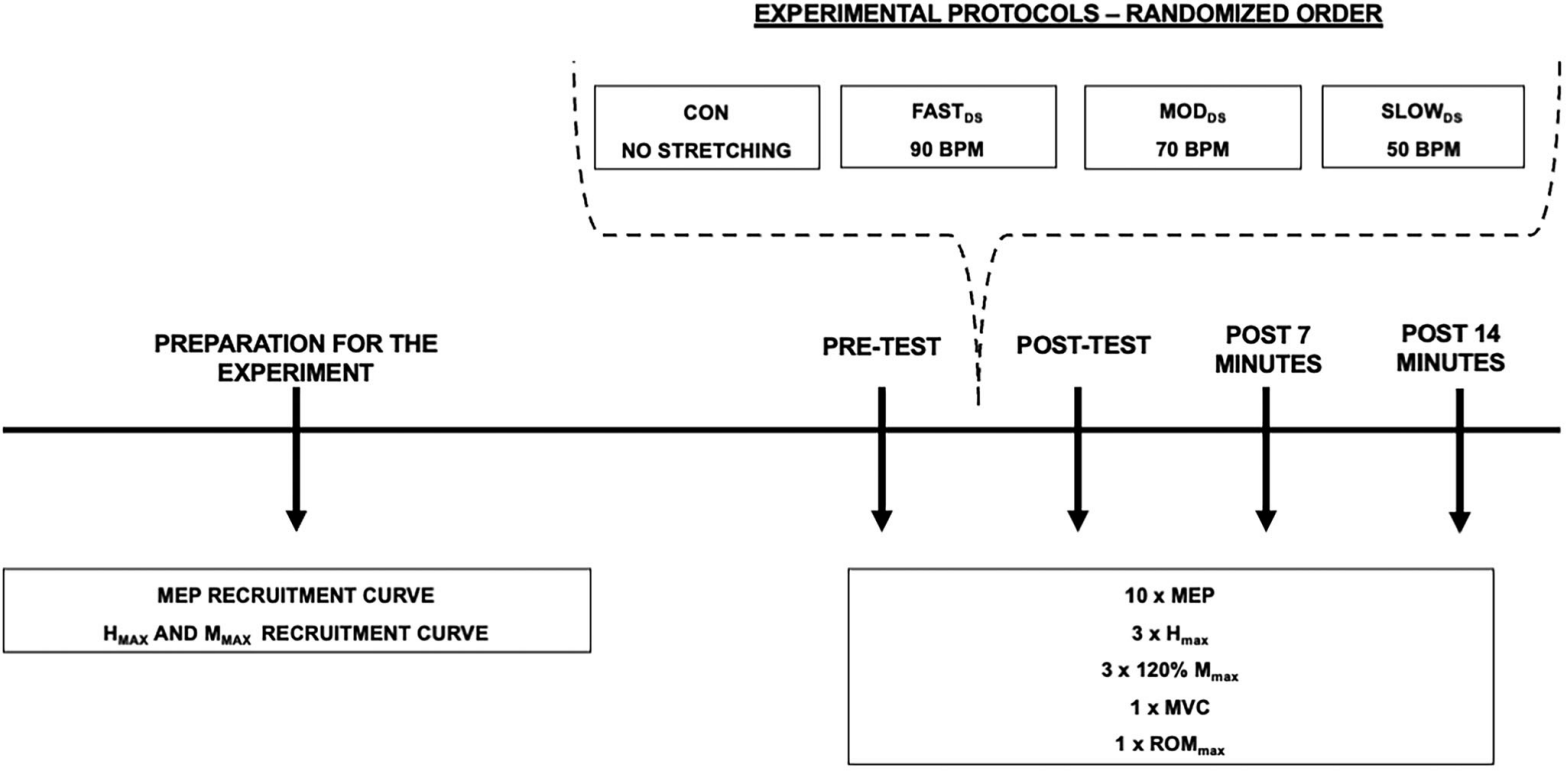
Experimental design. BPM, beats/min; CON, control session; FAST_DS_, dynamic stretching at fast velocity; *H*_max_, maximal H‐reflex; MEP, motor evoked potential; MOD_DS_, dynamic stretching at moderate velocity; *M*_max_, maximal M‐wave; MVC, maximal isometric voluntary contraction; ROM_max_, maximal range of motion; SLOW_DS_, dynamic stretching at slow velocity.

During all experimental sessions, no warm‐up was performed to avoid any influence on pre‐tests and to verify the effect of dynamic stretching as an isolated warm‐up component. Initially, the resting motor threshold of the soleus (SOL) muscle was identified from TMS, along with the maximal H‐reflex (*H*_max_) and M‐wave (*M*_max_) recruitment curves from tibial nerve electrical stimulation. Subsequently, participants remained at rest for 10 min. After this rest period, the pre‐tests (PRE) were conducted to determine corticospinal activity (motor‐evoked potential normalized by *M*_max_, that is, MEP/*M*_max_), spinal excitability (*H*_max_/*M*_max_), muscle‐evoked properties (*M*_max_ and peak twitch torque; PTT), and muscle performance (maximal isometric voluntary contraction (MVC) and maximal ROM). These test procedures were repeated immediately (POST), 7 min (P07) and 14 min (P14) after each experimental condition (Figure 1). To increase the interval between tests procedures and avoid successive assessments. The 7‐min rest interval between post interventions were chosen instead of the traditional 5‐min, as they lasted around 4 min.

All experimental procedures were completed on the right plantar flexor muscles using an isokinetic dynamometer (Biodex System 4, Biodex Corp., Shirley, NY, USA). To avoid gravitational influences, subjects lay on their left side. The left leg was flexed (about 90°) and the right leg was fully extended (0°) to ensure plantar flexor muscles were placed under significant stretch (Opplert & Babault, 2019). To minimize heel displacements, the foot was positioned and fastened inside a shoe, and firmly attached to the footplate of the dynamometer with straps. The lateral malleolus was aligned to the centre of rotation of the dynamometer. Volunteers' positioning was carefully registered and reproduced to permit consistent measurements during the different experimental sessions.

### 2.4 Electromyography

Electromyographic (EMG) activity was recorded from the soleus (SOL), gastrocnemius medialis (GM), gastrocnemius lateralis (GL) and tibialis anterior (TA) muscles of the right leg. The skin under the electrodes was shaved and cleaned with alcohol to ensure low impedance. EMG signals were recorded from one pair of silver chloride electrodes (10 mm diameter) with an inter‐electrode distance of 2 cm. The electrodes to record SOL EMG were placed 2 cm below the intersection over the Achilles tendon. For GM and GL EMG, the electrodes were placed over the mid‐belly of the muscles. TA electrodes were placed at one‐third of the distance between the lateral epicondyle of the tibia and the medial malleolus. The reference electrode was positioned over the contralateral patella (Opplert et al., 2020; Pulverenti et al., 2020). The EMG signal was amplified with a bandwidth frequency range from 10 Hz to 5 kHz (gain = 1000). EMG traces were recorded using a Biopac MP 150 system (Biopac Systems, Goleta, CA, USA) at a sampling rate of 10 kHz and stored for analysis with AcqKnowledge software (ver. 4.2, Biopac Systems).

### 2.5 Transcranial magnetic stimulation

Single TMS pulses were used to evoke MEPs with the ankle in a neutral position to assess corticospinal excitability. A double‐cone coil connected to a magnetic stimulator (Magstim 2002, Magstim, Whitland, UK) was positioned over the contralateral primary motor cortex at the area of homunculus corresponding to the triceps surae muscles approximately 10 mm posterior and lateral over the cortex of the subject's head. The optimal coil position was defined as the site eliciting the largest mean of the three MEPs in SOL with a minimal TA MEP amplitude (<50% of SOL MEP amplitude). Once the optimal site was determined, it was registered and marked with adhesive tape to ensure a constant positioning of the coil throughout all the tests and the different experimental sessions (Opplert et al., 2020; Pulverenti et al., 2020). The lowest intensity that yields resting SOL MEP amplitude >50 µV was considered the resting motor threshold (Awiszus, 2003). TMS intensity for all subsequent measures was then set at 120% of the resting motor threshold (Pulverenti et al., 2020). For the PRE, POST, P07 and P14 tests, 10 stimuli were delivered to determine SOL, GM and GL MEP amplitudes. At each muscle, the mean amplitude obtained from each 10 MEPs was normalized by *M*_max_ to avoid influences from the peripherical changes (Pulverenti et al., 2021), and to determine SOL MEP/*M*_max_, GM MEP/*M*_max_ and GL MEP/*M*_max_.

### 2.6 Neuromuscular electrical stimulation

The posterior tibial nerve was stimulated with rectangular pulses (1 ms) using a Digitimer stimulator (DS7A, Digitimer, Welwyn Garden City, UK) with the ankle in a neutral position. The cathode (10 mm diameter) was placed in the popliteal fossa and the anode (5 × 10 cm) over the patella on the anterior surface of the knee. The optimal stimulation site was determined by eliciting the greatest M‐wave for a given intensity with a hand‐held stimulation probe. Then, the stimulation electrode was pasted to the site (Vieira et al., 2021).

The recruitment curve was performed with stimulation intensity increasing in 2 mA increments from 0 mA to the SOL maximal peak‐to‐peak H‐reflex amplitude (*H*_max_). After reaching *H*_max_, 5 mA increments were used until SOL maximal peak‐to‐peak M‐wave amplitude (*M*_max_). For the PRE, POST, P07 and P14 tests, three electrical stimuli were delivered at an intensity relative to the SOL *H*_max_, and then, three electrical stimuli at an intensity relative to 120% of the SOL *M*_max_. Stimulations were administered with a 10 s rest interval (Aagaard et al., 2002; Opplert et al., 2020).

The mean H‐reflex and M‐wave amplitude of the SOL, GM and GL obtained from the three stimuli at each intensity relative to the SOL *H*_max_ and SOL *M*_max_ were considered as *H*_max_ and *M*_max_ of each muscle (Opplert et al., 2020). Then, the *H*_max_ was normalized by *M*_max_ (*H*_max_/*M*_max_) to avoid influences from the peripheral changes (Pulverenti et al., 2021). Additionally, torque was registered during the three electrical stimuli at an intensity relative to 120% of the *M*_max_, and the mean torque was considered for PTT analysis (Opplert & Babault, 2019; Opplert et al., 2020; Vieira et al., 2021).

### 2.7 Maximal voluntary contraction

A single isometric plantar flexor MVC with the ankle at a neutral position was performed to determine the maximal voluntary peak torque. The individuals were encouraged verbally to perform the maximal force during MVC, and the peak torque during MVC was registered and considered for analysis (Opplert & Babault, 2019; Opplert et al., 2020; Vieira et al., 2021).

### 2.8 Range of motion and passive torque

The maximal ROM (ROM_max_) and passive torque (PT) were measured during a passive stretch using the isokinetic dynamometer (Biodex System 4, Biodex Corp.). The participant's ankle was passively rotated from the neutral position until the maximal passive dorsiflexion at slow velocity (5°/s) to avoid the stretch reflex (Blazevich et al., 2014; Opplert & Babault, 2019). The ankle angle and PT were registered. ROM_max_ was the maximal ankle position relative to maximal passive dorsiflexion. Additionally, ΔPT was calculated as the torque variation during the last 5° until ROM_max_ (Opplert & Babault, 2019; Vieira et al., 2021).

### 2.9 Experimental conditions

During dynamic stretching, the participants were instructed to pull their right foot towards maximal dorsiflexion against the resistance provided by the mechanical inertia of the isokinetic dynamometer and to extend their foot back towards maximal plantar flexion. They were instructed to follow a rhythm controlled by a metronome to control movement velocity during dynamic stretching (MetroTimer 3.3.2, Onyx 3 Apps, Sofia, Bulgaria). The movement frequencies were 90, 70 or 50 beats/min for FAST_DS_, MOD_DS_ and SLOW_DS_, respectively. The dynamic stretching volume was four sets of 10 repetitions for all stretching protocols and the rest interval between the sets was 30 s (Mizuno, 2022). During the control session, the individuals were instructed to rest while lying for 5 min with the ankle in a neutral position. The control condition was included to verify if neuromuscular changes came from test repetition rather than dynamic stretches.

### 2.10 Statistical analysis

The absolute values and relative changes were reported as means ± SD. The Shapiro–Wilk test was used to verify the normality of data. A logarithm transformation was performed on non‐normality data (SOL‐MEP/*M*_MAX_ and GL‐MEP/*M*_max_). A two‐way (condition × time) repeated‐measures analysis of variance was used on absolute values and relative changes. The conditions corresponded to FAST_DS_, MOD_DS_, SLOW_DS_ and CON. For absolute values, the time at each experimental condition was PRE, POST, P07 and P14 tests. For relative changes, the time corresponded to percentage differences POST − PRE (Δ_POST_), P07 − PRE (Δ_P07_) and P14 − PRE (Δ_P14_). The Bonferroni *post hoc* test was performed in case of significant main effects or interactions. The level of significance for all comparisons was set at *P* < 0.05. Additionally, Cohen's *d* qualitative descriptors of standardized effect size were used for pair‐wise comparisons, such that values <0.2, 0.21–0.5, 0.51–1.2 and >1.2 represented trivial, small, moderate and large effect sizes, respectively (Cohen, 1988). All statistical procedures were performed in JASP (version 0.14, JASP Team 2020, University of Amsterdam; http://jasp‐stats.org/download).

## 3 RESULTS

Twenty‐three individuals were invited and agreed to participate in this study. However, five individuals were not included after answering the TMS readiness and reporting a history of concussion (*n* = 4) or epilepsy (*n* = 1). Additionally, four individuals were excluded due to reported headaches after the familiarization session. Thus, 14 individuals completed all experimental sessions. Nonetheless, the statistical analyses for MEP/*M*_max_, *H*_max_/*M*_max_ and *M*_max_ from SOL, GM and GL were performed with 13 individuals because the *M*_max_ data acquisition of one participant at each muscle showed signal saturation. The statistical analyses for MVC, PTT, ROM and ΔPT were done with the 14 individuals who completed all experimental sessions (Supporting information, Tables S1–S6).

**FIGURE 2:**
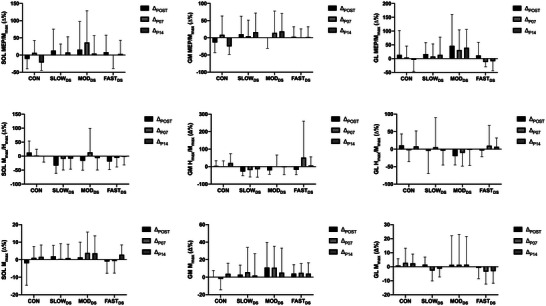
Relative Changes of the MEP/*M*_max_, *H*_max_/*M*_max_, and *M*_max_ from SOL, GM and GL. The data are reported in means and SD; *n* = 13 individuals. CON, control session; Δ_P07_, P07 relative changes according to PRE; Δ_P14_, P14 relative changes according to PRE; Δ_POST_, POST relative changes according to PRE; FAST_DS_, dynamic stretching at fast velocity; GL, gastrocnemius lateralis; GM, gastrocnemius medialis; *H*_max_/*M*_max_, maximal H‐reflex normalized by maximal M‐wave; MEP/*M*_max_, motor evoked potential normalized by maximal M‐wave; *M*_max_, maximal M‐wave; MOD_DS_, dynamic stretching at moderate velocity; SLOW_DS_, dynamic stretching at slow velocity; SOL, soleus.

For MEP/*M*_max_ from SOL, GM and GL (Table 1), there was no effect for time (*P* = 0.396, 0.684 and 0.653, respectively) or condition (*P* = 0.849, 0.562 and 0.699, respectively), nor condition × time interaction (*P* = 0.484, 0.407 and 0.522, respectively). Similarly, for MEP/*M*_max_ relative changes from SOL, GM and GL (Figure 2), there was no effect for time (*P* = 0.276, 0.482 and 0.597, respectively) or condition (*P* = 0.643, 0.371 and 0.247, respectively), nor condition × time interaction (*P* = 0.344, 0.159 and 0.541, respectively). For *M*_max_ absolute values from SOL, GM and GL, there was no effect for time (*P* = 0.239, 0.189 and 0.426, respectively) or condition (*P* = 0.442, 0.775 and 0.461, respectively), nor condition × time interaction (*P* = 0.390, 0.639 and 0.462, respectively). Additionally, for *M*_max_ relative changes from SOL, GM and GL, there was no effect for time (*P* = 0.077, 0.800 and 0.565, respectively) or condition (*P* = 0.691, 0.582 and 0.401, respectively), nor condition × time interaction (*P* = 0.161, 0.364 and 0.399, respectively).

**TABLE 1:** MEP/*M*_max_, *H*_max_/*M*_max_ and *M*_max_ absolute values from SOL, GM and GL.

		SOL			GM			GL		
		MEP/*M*_max_ (mV)	*H*_max_/*M*_max_ (mV)	*M*_max_ (mV)	MEP/*M*_max_ (mV)	*H*_max_/*M*_max_ (mV)	M_max_ (mV)	MEP/*M*_max_ (mV)	*H*_max_/*M*_max_ (mV)	*M*_max_ (mV)
CON	PRE	0.019 ± 0.016	0.41 ± 0.30	11.2 ± 4.3	0.018 ± 0.011	0.18 ± 0.16	7.7 ± 2.4	0.014 ± 0.019	0.16 ± 0.13	11.5 ± 4.5
	POST	0.014 ± 0.007	0.43 ± 0.27*	11.0 ± 4.6	0.014 ± 0.005	0.18 ± 0.14*	7.7 ± 2.5	0.011 ± 0.008	0.16 ± 0.14	11.6 ± 4.5
	P07	0.020 ± 0.017	0.38 ± 0.26	11.4 ± 4.5	0.019 ± 0.012	0.19 ± 0.21	7.6 ± 2.6	0.013 ± 0.014	0.14 ± 0.11	11.6 ± 4.4
	P14	0.014 ± 0.008	0.38 ± 0.26	11.5 ± 4.6	0.013 ± 0.007	0.19 ± 0.14	8.0 ± 2.5	0.011 ± 0.009	0.16 ± 0.14	11.7 ± 4.4
SLOW_DS_	PRE	0.016 ± 0.008	0.44 ± 0.32	10.5 ± 3.9	0.019 ± 0.012	0.21 ± 0.18	8.2 ± 3.9	0.013 ± 0.012	0.15 ± 0.12	10.6 ± 4.0
	POST	0.017 ± 0.011	0.32 ± 0.25*	10.9 ± 4.5	0.020 ± 0.018	0.15 ± 0.14*	8.5 ± 4.3	0.015 ± 0.013	0.15 ± 0.16	10.7 ± 3.9
	P07	0.020 ± 0.017	0.39 ± 0.29	10.7 ± 4.3	0.020 ± 0.019	0.15 ± 0.14	8.7 ± 4.6	0.016 ± 0.021	0.15 ± 0.13	10.3 ± 4.0
	P14	0.014 ± 0.008	0.28 ± 0.27	10.7 ± 4.4	0.022 ± 0.022	0.17 ± 0.16	8.3 ± 4.3	0.017 ± 0.022	0.14 ± 0.10	10.4 ± 4.0
MOD_DS_	PRE	0.016 ± 0.015	0.36 ± 0.24	12.0 ± 4.7	0.020 ± 0.017	0.18 ± 0.13	72 ± 3.1	0.012 ± 0.013	0.14 ± 0.08	10.1 ± 2.7
	POST	0.018 ± 0.018	0.32 ± 0.21*	12.0 ± 4.7	0.019 ± 0.012	0.14 ± 0.12*	7.8 ± 3.2	0.014 ± 0.009	0.12 ± 0.09	10.2 ± 3.0
	P07	0.022 ± 0.023	0.32 ± 0.17	12.2 ± 4.7	0.020 ± 0.013	0.15 ± 0.11	7.8 ± 3.2	0.014 ± 0.011	0.13 ± 0.11	10.2 ± 3.0
	P14	0.016 ± 0.019	0.32 ± 0.21	12.3 ± 4.8	0.022 ± 0.013	0.17 ± 0.17	7.5 ± 3.3	0.014 ± 0.011	0.14 ± 0.12	10.2 ± 3.0
FAST_DS_	PRE	0.018 ± 0.019	0.41 ± 0.26	11.7 ± 5.5	0.018 ± 0.010	0.20 ± 0.20	7.4 ± 2.7	0.012 ± 0.008	0.16 ± 0.14	10.2 ± 4.3
	POST	0.018 ± 0.015	0.35 ± 0.29*	11.7 ± 5.6	0.018 ± 0.012	0.17 ± 0.18*	7.8 ± 3.1	0.013 ± 0.009	0.15 ± 0.14	10.2 ± 4.5
	P07	0.016 ± 0.015	0.39 ± 0.28	11.7 ± 5.6	0.017 ± 0.010	0.22 ± 0.22	7.8 ± 2.9	0.010 ± 0.007	0.16 ± 0.14	9.9 ± 4.3
	P14	0.016 ± 0.013	0.40 ± 0.27	12.0 ± 5.6	0.017 ± 0.011	0.19 ± 0.20	7.6 ± 2.6	0.010 ± 0.007	0.15 ± 0.12	9.7 ± 3.4

The data are reported in means and SD; *n* = 13 individuals. *Statistical analyses indicate a significant main effect for time with *post hoc* Bonferroni test reporting a significant difference compared to the PRE moment within the same session (*P* = 0.017 for SOL, and *P* = 0.021 for GM). CON, control session; FAST_DS_, dynamic stretching performed in a fast velocity; GL, gastrocnemius lateralis; GM, gastrocnemius medialis; *H*_max_/*M*_max_, maximal H‐reflex normalized by maximal M‐wave; MEP/*M*_max_, motor evoked potential normalized by maximal M‐wave; *M*_max_, maximal M‐wave; MOD_DS_, dynamic stretching performed in a moderate velocity; P07, test performed 7 min after experimental condition; P14, test performed 14 min after experimental condition; POST, test performed immediately after experimental condition; PRE, Pre‐test; SLOW_DS_, dynamic stretching performed in a slow velocity; SOL, soleus.

For *H*_max_/*M*_max_ absolute values from SOL and GM, there was no effect for condition (*P* = 0.390 and 0.649, respectively), nor condition × time interaction (*P* = 0.091 and 0.441, respectively). However, there was a main effect for time (0.048 and 0.047, respectively) with a Bonferroni *post hoc* test reporting a significant difference between PRE and POST (*P* = 0.017 for SOL, and *P* = 0.021 for GM). Cohen's *d* value reported a trivial or a small clinical decrease of the SOL *H*_max_/*M*_max_ (0.47, 0.16 and 0.23 for SLOW_DS_, MOD_DS_ and FAST_DS_, respectively) and GM *H*_max_/*M*_max_ (0.37, 0.24 and 0.18 for SLOW_DS_, MOD_DS_ and FAST_DS_, respectively) immediately after the dynamic stretching sessions, regardless of the movement velocity. Conversely, immediately after the control session, Cohen's *d* value reported no clinical changes in the SOL *H*_max_/*M*_max_ (−0.06 for CON) and GM *H*_max_/*M*_max_ (0.03 for CON). For relative changes in *H*_max_/*M*_max_ for SOL, GM and GL, there was no effect for time (*P* = 0.118, 0.102 and 0.565, respectively) or condition (*P* = 0.238, 0.333 and 0.401, respectively), nor condition × time interaction (*P* = 0.142, 0.329 and 0.399, respectively).

**FIGURE 3:**
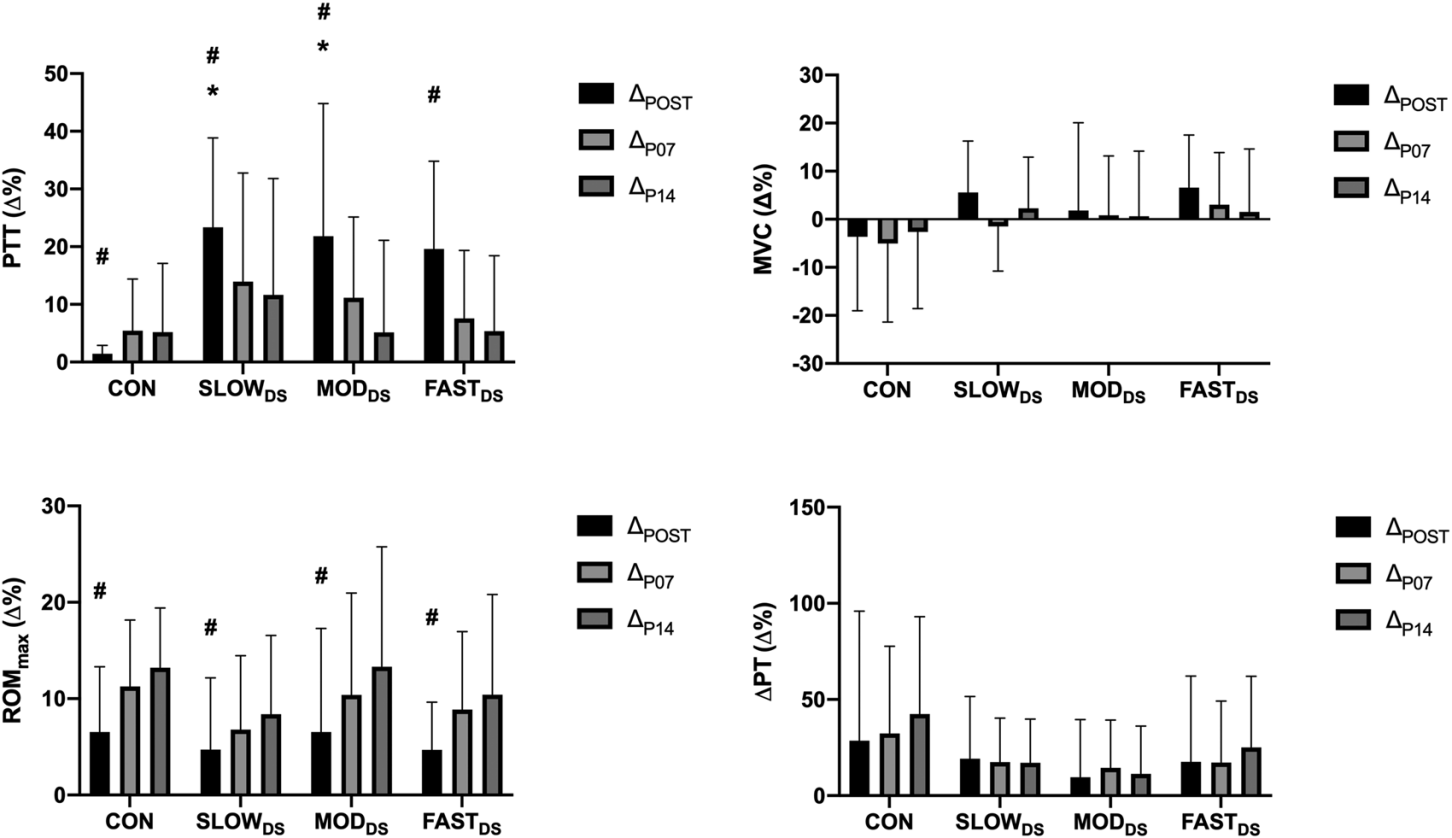
Relative PTT, MVC, ROM_max_, and ΔPT changes. The data are reported in means and SD; *n* = 14 individuals. #Significant differences with Δ_P07_ and Δ_P14_ within the same session (*P *< 0.05). *Significant differences with the CON for the same time point (*P < *0.05). CON, control session; Δ_P07_, P07 relative changes according to PRE; Δ_P14_, P14 relative changes according to PRE; Δ_POST_, POST relative changes according to PRE; ΔPT, difference in passive torque from maximal range of motion and 5° less than maximal range of motion; FAST_DS_, dynamic stretching performed in a fast velocity; MOD_DS_, dynamic stretching performed in a moderate velocity; MVC, maximal voluntary contraction; PTT, peak twitch torque; ROM_max_, maximal range of motion; SLOW_DS_, dynamic stretching performed in a slow velocity.

For PTT absolute values and relative changes (Table 2 and Figure 3, respectively), there was no significant main effect for condition (*P* = 0.414, and 0.081, respectively) but a significant main effect for time (*P* < 0.001 for both absolute values and relative changes), and time × condition interaction (*P* = 0.002 for both absolute values and relative changes). As compared to PRE, *post hoc* analyses showed that PTT was greater in the POST after SLOW_DS_ (*P* < 0.001), MOD_DS_ (*P* < 0.001) and FAST_DS_ (*P* < 0.001). Additionally, PTT at P07 was greater than PRE just in SLOW_DS_ (*P* = 0.047), and MOD_DS_ (*P* = 0.045). Cohen's *d* value of the absolute PTT reported moderate increase immediately after all stretching conditions (−0.62, −0.65 and −0.58 for SLOW_DS_, MOD_DS_ and FAST_DS_, respectively). Conversely, immediately after the control session, Cohen's *d* value reported no clinical changes in the PTT (−0.04 for CON). The PTT relative changes at Δ_POST_ was significantly greater in SLOW_DS_ (*P* = 0.005) and MOD_DS_ (*P* = 0.014) than in CON. Although *post hoc* analyses report that FAST_DS_ was not significantly greater than CON at Δ_POST_, it was close to the statistical significance (*P* = 0.057). Moreover, the *post hoc* analyses for the main time effect reported that PTT relative changes at Δ_POST_ were greater than Δ_P07_ (*P* = 0.04) and Δ_P14_ (*P* < 0.001).

**TABLE 2:** PTT, MVC, ROM_max_ and ΔPT absolute values.

		PTT (N m)	MVC (N m)	ROM_max_ (°)	ΔPT (N m)
CON	PRE	13.3 ± 3.4	105.2 ± 32.6	40.0 ± 6.4	12.4 ± 6.6
	POST	13.5 ± 3.5	100.3 ± 30.6	42.5 ± 6.2*	14.2 ± 6.8*
	P07	14.0 ± 3.9	99.7 ± 33.6	44.3 ± 6.5*^#^	15.0 ± 6.6*
	P14	13.9 ± 3.7	101.9 ± 32.1	45.1 ± 6.6*^#^	16.3 ± 7.5*
SLOW_DS_	PRE	13.0 ± 4.1	106.5 ± 41.0	40.3 ± 5.8	13.4 ± 5.3
	POST	15.6 ± 4.0*	110.5 ± 39.4	42.1 ± 5.9*	15.3 ± 6.4*
	P07	14.5 ± 4.0*	103.0 ± 35.3	42.9 ± 5.8*^#^	15.4 ± 6.1*
	P14	14.1 ± 3.8	106.7 ± 36.6	43.5 ± 5.8*^#^	14.9 ± 4.6*
MOD_DS_	PRE	13.2 ± 4.0	105.0 ± 31.3	41.3 ± 6.1	13.4 ± 5.1
	POST	16.0 ± 5.0*	107.2 ± 37.9	43.7 ± 5.6*	15.0 ± 7.1*
	P07	14.7 ± 4.7*	107.2 ± 38.8	45.2 ± 5.1*^#^	15.4 ± 7.0*
	P14	13.9 ± 4.4	106.4 ± 38.3	46.2 ± 4.9*^#^	15.2 ± 6.8*
FAST_DS_	PRE	13.7 ± 4.3	101.7 ± 30.7	38.7 ± 5.9	12.9 ± 6.3
	POST	16.1 ± 4.6*	109.2 ± 36.6	41.6 ± 6.1*	14.0 ± 7.4*
	P07	14.7 ± 4.9	105.7 ± 36.8	43.1 ± 6.0*^#^	14.4 ± 6.9*
	P14	14.3 ± 4.5	103.4 ± 33.7	44.0 ± 8.1*^#^	15.0 ± 6.7*

The data are reported in mean and SD; *n* = 14 individuals. *Significant differences with the PRE within the same session (*P *< 0.05). **#** Significant differences with the POST within the same session (*P *< 0.05). CON, control session; ΔPT, difference in passive torque from maximal range of motion and 5° less than maximal range of motion; FAST_DS_ = dynamic stretching performed in a fast velocity; MOD_DS_, dynamic stretching performed in a moderate velocity; MVC, maximal isometric voluntary contraction; P07, test performed 7 min after experimental condition; P14, test performed 14 min after experimental condition; PTT, peak twitch torque; PRE, pre‐test; POST, test performed immediately after experimental condition; ROM_max_, maximal range of motion; SLOW_DS_, dynamic stretching performed in a slow velocity.

For MVC absolute values and relative changes and ΔPT relative changes, there was no main effect for time (*P = *0.417, 0.101 and 0.262, respectively) or condition (*P* = 0.507, 0.281 and 0.413, respectively), nor condition × time interaction (*P* = 0.250, 0.608 and 0.440, respectively). Cohen's *d* of the MVC absolute values reported a trivial or small increase immediately after all stretching conditions (−0.11, −0.06 and −0.21 for SLOW_DS_, MOD_DS_ and FAST_DS_, respectively). Conversely, immediately after the control session, Cohen's *d* value reported a trivial decrease in the MVC (0.14 for CON).

For ROM_max_ absolute values and relative changes and ΔPT absolute values, there was neither a main effect for condition (*P* = 0.099, 0.474 and 0.805, respectively), nor condition × time interaction (*P* = 0.708, 0.788 and 0.525, respectively). However, there was a significant main effect for time (*P* < 0.001 for ROM_max_ absolute values and relative changes, and ΔPT absolute values). The *post hoc* analyses revealed that ROM_max_ and ΔPT absolute values were greater in POST (*P* < 0.001 and *P* = 0.004, respectively), P07 (*P* < 0.001 for both ROM_max_ and ΔPT absolute values), and P14 (*P* < 0.001 for both ROM_max_ and ΔPT absolute values) than in PRE. Additionally, just in ROM_max_ absolute values, P07 (*P* = 0.019) and P14 (*P* < 0.001) were greater than POST, but no difference was found between P07 and P14 (*P* = 0.395). The *post hoc* analyses showed that relative changes in ROM_max_ at Δ_POST_ were lower than Δ_P07_ (*P* < 0.001), and Δ_P14_ (*P* < 0.001). Cohen's *d* value reported a small increase in the ROM_max_ immediately after all conditions (−0.39, −0.28, −0.38 and −0.29 for CON, SLOW_DS_, MOD_DS_ and FAST_DS_, respectively). Cohen's *d* value reported a trivial to small increase in the ΔPT immediately after all conditions (−0.27, −0.28, −0.24 and −0.16 for CON, SLOW_DS_, MOD_DS_ and FAST_DS_, respectively).

## 4 DISCUSSION

This study verified the acute effects of dynamic stretching velocity on specific sites within the neuromuscular system (i.e., corticospinal excitability, spinal reflex activity, contractile properties and muscle stiffness) and muscle mechanisms (strength, stiffness, and ROM_max_). Additionally, the study verified the time course effects of dynamic stretching at various velocities on neuromuscular adaptations. The results indicated that dynamic stretching did not have effects on corticospinal excitability. However, it decreased spinal reflex activity, regardless of the movement velocity. Furthermore, dynamic stretching, regardless of movement velocity, increased evoked contractile properties, and it remained for several minutes. However, dynamic stretching did not enhance isometric force production. Finally, all dynamic stretching conditions increased ROM and PT. However, similar results were observed in the control session, suggesting that these ROM and PT adaptations might result from repeated testing rather than dynamic stretching.

Our study found no effect on SOL, GL and GM MEP/*M*_max_ after four sets of 10 repetitions of dynamic stretching, regardless of movement velocity. This finding is consistent with Guissard et al. (2001), who reported no impact on SOL MEP/*M*_max_ following passive dynamic stretching of the plantar flexor. While corticospinal excitability may change during the muscle lengthening in stretching movements, it appears to return to baseline values within a few seconds after the stretch ends (Budini et al., 2018; Guissard et al., 2001). This quick return to baseline could explain the absence of MEP/*M*_max_ changes in triceps surae muscles after dynamic stretching observed in our study.

Indeed, our corticospinal excitability assessment began approximately 10 s after completing the dynamic stretching protocols. We conducted the assessment using 10 MEP assessments over around 2 min. Although a higher number of MEPs can increase test reliability, it also lengthens the measurement period (Pulverenti et al., 2019). Thus, the duration of MEP assessment in our study might have been too long to detect any transient effect of the stretching. Different results might be obtained with fewer MEPs. For example, Opplert et al. (2020) used just six MEP assessments and found that GL MEP/*M*_max_ increased after five sets of 20 repetitions of plantar flexor dynamic stretching at 60 beats/min.

Besides the total duration of the MEP measurement, methodological differences might also explain the discrepancies between our results and those of Opplert et al. (2020). One key difference is muscle state (i.e., contracted or relaxed) during MEP assessment. In our study, MEP/*M*_max_ was evaluated while the muscle was at rest, whereas Opplert et al. (2020) conducted their assessments during a submaximal muscle contraction at 30% of MVC. They suggested that the impact of muscle stretching might be more pronounced when MEP/*M*_max_ assessments are performed with a background of submaximal voluntary contraction (Opplert et al., 2020). Indeed, the muscle state may affect MEP/*M*_max_ amplitude during corticospinal measurements (Gruet et al., 2013; Nielsen & Petersen, 1995).

Our results indicated that SOL *H*_max_/*M*_max_ and GL *H*_max_/*M*_max_ were reduced immediately after dynamic stretching, regardless of the velocity. Since *H*_max_/*M*_max_ is an index of spinal reflex activity and motor neuron excitability, this reduction suggests a decrease in the α‐motor neuron pool to the muscle following dynamic stretching (Aagaard et al., 2002). Typically, the amplitude of the H‐reflex is influenced by excitatory and inhibitory mechanisms in the α‐motor neurons (Clark et al., 2014; Stevanovic et al., 2019). For example, a reduction in H‐reflex could be due to a diminution in the excitatory drive from the Ia afferents to the α‐motor neurons (Avela et al., 1999). It has been proposed that muscle elongation from stretching may lead to desensitization of the muscle spindles’ proprioceptive structures, thereby affecting Ia afferent drive to the α‐motor neuron (Avela et al., 1999; Trajano et al., 2014). However, the adaptations of the muscle–tendon unit following dynamic stretching are primarily influenced by tendon unit displacement rather than muscle tissue (Vieira et al., 2021). Dynamic stretching elongates the muscle–tendon unit without altering the pennation angle and fascicle length of the muscle (Samukawa et al., 2011). Therefore, the decrease in Ia afferent excitatory drive to α‐motor neurons due to a muscle elongation, which could lead to desensitization of the muscle spindles, may not be the reason for the observed H‐reflex depression in our study.

Presynaptic inhibitory mechanisms, such as a reduction in neurotransmitter release from Ia afferents due to depletion of neurotransmitter vesicles or calcium channel inactivation, and γ‐motor efferent stimulation caused by stretch could decrease spinal reflex activity (Kohn et al., 1997; Taylor et al., 1985). However, Clark et al. (2014) previously reported that dynamic stretching reduces presynaptic inhibition. Therefore, contrary to our findings, an increase rather than a decrease in H‐reflex responses after dynamic stretching would be expected. The postsynaptic mechanism can also reduce spinal reflex activity and decrease H‐reflex responses (Stevanovic et al., 2019). Repetitive movements may increase inhibitory postsynaptic potentials from interneurons, leading to a hyperpolarization onto α‐motor neurons and postsynaptic receptor desensitization, which makes the receptors less responsive to the neurotransmitters (Guissard et al., 2001; Kohn et al., 1997). Since dynamic stretching involves repetitive cyclic movements of muscle lengthening and shortening (Opplert & Babault, 2019; Vieira et al., 2021), it could lead to these above‐mentioned inhibitory postsynaptic compensation mechanisms, that is, hyperpolarization onto α‐motor neurons and postsynaptic receptor desensitization. Therefore, it may help to explain the diminution of the *H*_max_/*M*_max_ amplitude observed in our study after dynamic stretching.

Our study found that dynamic stretching, regardless of movement velocity, increased PTT. This increase in PTT remained for several minutes, highlighting the importance of dynamic stretching for practical application in sports. Since peak performance in sports typically occurs shortly after a warm‐up (Pamboris et al., 2019), the prolonged effect of dynamic stretching on muscle contractile properties reported in our study may benefit athletes. The enhancements in evoked contractile properties may be partly attributed to temperature‐related mechanisms (Vieira et al., 2021). Dynamic stretching increases muscle temperature, which may improve muscle fibre conduction velocity and cross‐bridge cycling rate, thus enhancing the muscle contractile properties (Bishop, 2003; Maffiuletti et al., 2016; McGowan et al., 2015).

However, the enhancement in PTT was not accompanied by an increase in MVC. This suggests that improvements in peripherical mechanisms, such as muscle contractile properties, from dynamic stretching may not enhance voluntary force. Neural mechanisms might also influence voluntary force effects from a bout of stretching (Opplert & Babault, 2018, 2019; Vieira et al., 2021). Previously, Vieira et al. (2021) found a decrease in the neural efferent drive to the muscle after plantar flexor dynamic stretching. Additionally, our present findings showed a reduced spinal reflex activity after plantar flexor dynamic stretching. Therefore, the lack of effect of dynamic stretching on MVC observed in our findings might result from the counterbalanced effects of neural impairments and peripherical muscle contractile improvements.

Lastly, our study found that all dynamic stretching conditions increased the range of motion and passive torque, which agrees with previous findings (Mizuno, 2022; Pamboris et al., 2019). However, these results should be interpreted cautiously because the previous studies did not include a control session (Mizuno, 2022; Pamboris et al., 2019). Moreover, our study observed a similar increase in range of motion and passive torque in the control session compared to all dynamic stretching conditions. Therefore, these increases may have resulted from repeated testing rather than dynamic stretching. Indeed, ROM_max_ assessment involves passive muscle stretches at maximal amplitudes leading to muscle creep. Additionally, muscle contraction induced by MEPs, H‐reflex, M‐wave and MVC assessments could lead to muscle warm‐up. Consequently, all repetitive tests potentially induce viscoelastic changes, leading to ROM_max_ and stiffness adaptations (Opplert & Babault, 2019; Vieira et al., 2021).

Some limitations should be mentioned, first, our study performed just one MVC assessment. Traditionally, the studies performed two assessments to verify the reproducibility of the measure and define the best MVC assessment (Pulverenti et al., 2019; Trajano et al., 2013). Thus, the lack of significance may be related to these methodological aspects, as the effect size reported a trivial to small increase in MVC after all stretching conditions and a trivial decrease after the control session. However, we chose just one assessment to reduce the time of the measures at post‐tests and avoid a warm‐up effect from multiple MVCs at the pre‐tests. Second, a priori sample size calculation based on a previous study reported that a sample size of 13 individuals was deemed enough. However, MEP is a highly variable measure, and therefore a larger sample size may be required. Thus, we cannot discard the hypothesis that the lack of significance in MEP/*M*_max_ may result from a lower number of participants.

### 4.1 Conclusion

The findings from our study indicate that corticospinal excitability was not adversely affected by dynamic stretching, irrespective of movement velocity. However, dynamic stretching may reduce spinal excitability. Importantly, dynamic stretching did not impair isometric muscle strength, as all dynamic stretching protocols did not affect MVC. Furthermore, regardless of movement velocity, dynamic stretching enhanced muscle contractile properties, and this effect persisted for several minutes, which is beneficial for practical applications in sports. The improvements in ROM_max_ and PT following dynamic stretching should be carefully interpreted, as the repeated tests may increase ROM_max_ and PT similarly to dynamic stretching. Lastly, our study contributes significantly to the literature on warm‐ups by demonstrating the acute effects of dynamic stretching on spinal reflex excitability and muscle contractile properties are independent of the dynamic stretching velocity.

## AUTHOR CONTRIBUTIONS

Conception and design of the work: Denis César Leite Vieira, Nicolas Babault and Martim Bottaro Acquisition, analysis or interpretation of the data for the work: Denis César Leite Vieira, Marion Hitier, João Luiz Quagliotti Durigan, Nicolas Babault and Martim Bottaro Drafting the work or revising it critically for important intellectual content: all authors. All authors have read and approved the final version of this manuscript and agree to be accountable for all aspects of the work in ensuring that questions related to the accuracy or integrity of any part of the work are appropriately investigated and resolved. All person designated as authors qualify for authorship, and all those who qualify for authorship are listed.

## CONFLICT OF INTEREST

The authors declare no competing interests.

## Supporting information



Table S1. Results of the two‐way repeated measures ANOVA for absolute values.
Table S2. Detailed results of the *post hoc* test with Bonferroni correction for the significant time effects for absolute values
Table S3. Detailed results of the *post hoc* test with Bonferroni correction for significant time × condition interaction for absolute values
Table S4. Results of the two‐way repeated measures ANOVA for relative changes.
Table S5. Detailed results of the *post hoc* test with Bonferroni correction for the significant time effects for relative changes.
Table S6. Detailed results of the *post hoc* test with Bonferroni correction for significant time × condition interaction for relative changes.

## Data Availability

Data are available upon reasonable request. Figures 2 and 3 with individual data are available at: https://doi.org/10.6084/m9.figshare.27643947
