# Supplementary material for: The acute effects of dynamic stretching on the neuromuscular system are independent of the velocity
Source: Exp Physiol. 2025 Jan 6;110(3):494–505. doi: 10.1113/EP092217 (PMC11868028; doi:10.1113/EP092217)
Supplement: Supplementary file 1 — Table S1. Results of the two‐way repeated measures ANOVA for absolute values. Table S2. Detailed results of the post hoc test with Bonferroni correction for the significant time effects for absolute values Table S3. Detailed results of the post hoc test with Bonferroni correction for significant time × condition interaction for absolute values Table S4. Results of the two‐way repeated measures ANOVA for relative changes. Table S5. Detailed results of the post hoc test with Bonferroni correction for the significant time effects for relative changes. Table S6. Detailed results of the post hoc test with Bonferroni correction for significant time × condition interaction for relative changes. [file EPH-110-494-s001.docx]

**Supplementary table 1.** Results of the two-way repeated measures ANOVA for absolute values.

|  | | Condition | | | Time | | | Condition x Time | | |
| --- | --- | --- | --- | --- | --- | --- | --- | --- | --- | --- |
| Variable | Muscle | F | p | pη^2^ | F | p | pη^2^ | F | p | pη^2^ |
| MEP/M_max_ | SOL | 0.26 | 0.849 | 0.02 | 1.01 | 0.396 | 0.07 | 0.95 | 0.484 | 0.07 |
|  | GM | 0.69 | 0.562 | 0.05 | 0.50 | 0.684 | 0.04 | 1.01 | 0.407 | 0.07 |
|  | GL | 0.47 | 0.699 | 0.03 | 0.54 | 0.653 | 0.04 | 0.90 | 0.522 | 0.07 |
| H_max_/M_max_ | SOL | 1.03 | 0.390 | 0.07 | 3.63 | 0.048 | 0.23 | 2.05 | 0.091 | 0.14 |
|  | GM | 0.38 | 0.649 | 0.03 | 3.30 | 0.031* | 0.21 | 0.94 | 0.441 | 0.07 |
|  | GL | 0.13 | 0.936 | 0.01 | 0.19 | 0.899 | 0.01 | 0.37 | 0.814 | 0.03 |
| M_max_ | SOL | 0.82 | 0.442 | 0.06 | 1.52 | 0.239 | 0.11 | 1.04 | 0.390 | 0.08 |
|  | GM | 0.37 | 0.775 | 0.03 | 1.67 | 0.189 | 0.12 | 0.59 | 0.639 | 0.04 |
|  | GL | 0.88 | 0.461 | 0.06 | 0.95 | 0.426 | 0.07 | 0.88 | 0.462 | 0.06 |
| PTT | - | 0.97 | 0.414 | 0.07 | 21.46 | 0.001* | 0.62 | 4.87 | 0.002 | 0.27 |
| MVC | - | 0.79 | 0.507 | 0.05 | 0.89 | 0.417 | 0.06 | 1.29 | 0.25 | 0.09 |
| ROM_max_ | - | 2.23 | 0.099 | 0.14 | 35.79 | 0.001* | 0.73 | 0.60 | 0.708 | 0.04 |
| ΔPT | - | 0.32 | 0.805 | 0.02 | 11.68 | 0.001* | 0.47 | 0.80 | 0.525 | 0.05 |

MEP/M_MAX_ = Motor evoked potential normalized by maximal M_WAVE_; H_max_/M_MAX_ = Maximal H_REFLEX_ normalized by maximal M_WAVE_; M_MAX_ = Maximal M_WAVE_; SOL = Soleus; GM = Gastrocnemius Medialis; GL = Gastrocnemius Lateralis; PTT = Peak Twitch Torque; MVC = Maximal Isometric Voluntary Contraction; ROM_MAX_ = Maximal Range of Motion; ΔPT = Difference in passive torque from Maximal Range of Motion and 5° less than Maximal Range of Motion; F = F ratio; p = level of significance; pη^2^ = Partial Etta Square.* significant time effect.

**Supplementary table 2.** Detailed results of the post-hoc with Bonferroni corrections for the significant time effects for absolute values

|  | | PRE vs. POST | | | PRE vs. P07 | | | PRE vs. P14 | | |
| --- | --- | --- | --- | --- | --- | --- | --- | --- | --- | --- |
| Variable | Muscle | Mean difference  (95% CI) | d | p | Mean difference  (95% CI) | d | p | Mean difference  (95% CI) | d | p |
| H_max_/M_max_ | SOL | 0.053 (0.007; 0.098) | 0.198 | 0.017* | 0.036 (-0.010; 0.081) | 0.016 | 0.134 | 0.034 (-0.011; 0.080) | 0.129 | 0.260 |
|  | GM | 0.035 (0.004; 0.067) | 0.207 | 0.021* | 0.017 (-0.015; 0.048) | 0.097 | 0.914 | 0.014 (-0.018; 0.046) | 0.083 | 1.000 |
| PTT | - | -2.108 (-3.086; -0.950) | -0.475 | 0.001* | -1.184 (-2.027; -0.341) | -0.278 | 0.005* | -0.761 (-1.515; -0.008) | -0.179 | 0.047 |
| ROM_max_ | - | -2.095 (-3.350; -0.839) | -0.342 | 0.001* | -3.516 (-4.772; -2.261) | -0.574 | 0.001* | -4.371 (-5.627; -3.116) | -0.713 | 0.001* |
| ΔPT | - | -1.576 (-2.758; -0.393) | -0.241 | 0.004* | -2.007 (-3.190; -0824) | -0.307 | 0.001* | -2.315 (-3.489; 1.132) | -0.354 | 0.001* |

H_max_/M_MAX_ = Maximal H_REFLEX_ normalized by maximal M_WAVE_; M_MAX_ = Maximal M_WAVE_; SOL = Soleus; GM = Gastrocnemius Medialis; PTT = Peak Twitch Torque; ROM_MAX_ = Maximal Range of Motion; ΔPT = Difference in passive torque from Maximal Range of Motion and 5° less than Maximal Range of Motion; PRE = Pre-test; POST = test performed immediately after experimental condition; P07 = test performed 7 minutes after experimental condition; P14 = test performed 14 minutes after experimental condition; CI = Confidence Interval; d = Cohen’s D; p = level of significance. * significant time effect.

**Supplementary table 3.** Detailed results of the post-hoc with Bonferroni corrections for significant time x condition interaction for absolute values

|  | | PRE vs. POST | | | PRE vs. P07 | | | PRE vs. P14 | | |
| --- | --- | --- | --- | --- | --- | --- | --- | --- | --- | --- |
| Variable | Condition | Mean difference  (95% CI) | d | p | Mean difference  (95% CI) | d | p | Mean difference  (95% CI) | d | p |
| PTT | CON | -0.184 (-1.661; 1.293) | -0.043 | 1.000 | -0.729 (-2.206; 0.749) | -0.171 | 1.000 | -0.600 (-2.077; 0.877) | -0.141 | 1.000 |
|  | SLOW_DS_ | -2.645 (-4.123; -1.168) | -0.622 | 0.001* | -1.484 (2.961; -0.006) | -0.349 | 0.047* | -1.122 (-2.600; 0.355) | -0.264 | 0.817 |
|  | MOD_DS_ | -2.763 (-4.240; -1.285) | 0.409 | 0.001* | -1.490 (-2.967; -0.013) | -0.350 | 0.045* | -0.700 (-2.178; 0.777) | -0.165 | 1.000 |
|  | FAST_DS_ | -2.481 (-3.958; -1.003) | -0.583 | 0.001* | -1.034 (-2.511; 0.444) | -0.243 | 1.000 | -0.623 (-2.100; 0.854) | -0.146 | 1.000 |

PTT = Peak Twitch Torque; CON = Control Session; SLOW_DS_ = Dynamic stretching performed at a slow velocity; MOD_DS_ = Dynamic stretching performed at a moderate velocity; FAST_DS_ = Dynamic stretching performed at a fast velocity; PRE = Pre-test; POST = test performed immediately after experimental condition; P07 = test performed 7 minutes after experimental condition; P14 = test performed 14 minutes after experimental condition; CI = Confidence Interval; d = Cohen’s D; p = level of significance.* significant difference from CON.

**Supplementary table 4.** Results of the two-way repeated measures ANOVA for relative changes.

|  | | Condition | | | Time | | | Condition x Time | | |
| --- | --- | --- | --- | --- | --- | --- | --- | --- | --- | --- |
| Variable | Muscle | F | p | pη^2^ | F | p | pη^2^ | F | p | pη^2^ |
| MEP/M_max_ | SOL | 0.56 | 0.643 | 0.04 | 1.35 | 0.276 | 0.102 | 1.15 | 0.344 | 0.08 |
|  | GM | 1.07 | 0.371 | 0.08 | 0.75 | 0.482 | 0.05 | 1.80 | 0.159 | 0.13 |
|  | GL | 1.44 | 0.247 | 0.10 | 0.52 | 0.597 | 0.04 | 0.84 | 0.541 | 0.06 |
| H_max_/M_max_ | SOL | 1.47 | 0.238 | 0.10 | 2.34 | 0.118 | 0.16 | 2.05 | 0.142 | 0.14 |
|  | GM | 1.10 | 0.333 | 0.08 | 2.86 | 0.102 | 0.19 | 1.13 | 0.329 | 0.086 |
|  | GL | 0.43 | 0.727 | 0.03 | 0.51 | 0.603 | 0.04 | 0.64 | 0.693 | 0.05 |
| M_max_ | SOL | 0.36 | 0.691 | 0.02 | 2.85 | 0.077 | 0.19 | 1.84 | 0.161 | 0.13 |
|  | GM | 0.51 | 0.580 | 0.04 | 0.22 | 0.800 | 0.01 | 1.11 | 0.364 | 0.08 |
|  | GL | 0.45 | 0.565 | 0.03 | 0.94 | 0.401 | 0.07 | 1.01 | 0.399 | 0.07 |
| PTT | - | 2.42 | 0.081 | 0.15 | 13.50 | 0.001* | 0.51 | 7.05 | 0.002 | 0.35 |
| MVC | - | 1.32 | 0.281 | 0.09 | 2.50 | 0.101 | 0.16 | 0.75 | 0.608 | 0.05 |
| ROM_max_ | - | 0.85 | 0.474 | 0.06 | 21.10 | 0.001* | 0.61 | 0.39 | 0.788 | 0.03 |
| ΔPT | - | 0.32 | 0.805 | 0.02 | 11.68 | 0.001* | 0.47 | 0.80 | 0.520 | 0.05 |

MEP/M_MAX_ = Motor evoked potential normalized by maximal M_WAVE_; H_max_/M_MAX_ = Maximal H_REFLEX_ normalized by maximal M_WAVE_; M_MAX_ = Maximal M_WAVE_; SOL = Soleus; GM = Gastrocnemius Medialis; GL = Gastrocnemius Lateralis; PTT = Peak Twitch Torque; MVC = Maximal Isometric Voluntary Contraction; ROM_MAX_ = Maximal Range of Motion; ΔPT = Difference in passive torque from Maximal Range of Motion and 5° less than Maximal Range of Motion; F = F ratio; p = level of significance; pη^2^ = Partial Etta Square. * significant time effect.

**Supplementary table 5.** Detailed results of the post-hoc with Bonferroni corrections for the significant time effects for relative changes.

|  | Δ_POST_ vs. Δ_P07_ | | | Δ_POST_ vs. Δ_P14_ | | | Δ_P07_ vs. Δ_P14_ | |  |
| --- | --- | --- | --- | --- | --- | --- | --- | --- | --- |
| Variable | Mean difference  (95% CI) | d | p | Mean difference  (95% CI) | d | p | Mean difference  (95% CI) | d | p |
| PTT | 7.026 (2.087; 11.966) | 0.460 | 0.004 | -9.714 (-4.775; 14.6541) | 0.636 | 0.001* | 2.688 (-2.525; 7.627) | 0.176 | 0.527 |
| ROM_max_ | -3.707 (-5.991; -1.423) | -0.430 | 0.001* | -5.716 (-8.000; -3.432) | -0.662 | 0.001* | -2.009 (-4.293; -0.275) | -0.233 | 0.099 |
| ΔPT | -1.610 (-9.744; 6.524) | -0.042 | 1.000 | -5.212 (-13.346; 2.922) | -0.135 | 0.339 | -3.602 (-11.736; 4.532) | -0.094 | 0.802 |

Δ_POST_ = POST Relative changes according to PRE; Δ_P07_ = P07 Relative changes according to PRE; Δ_P14_ = P14 Relative changes according to PRE; PTT = Peak Twitch Torque; ROM_MAX_ = Maximal Range of Motion; ΔPT = Difference in passive torque from Maximal Range of Motion and 5° less than Maximal Range of Motion; CI = Confidence Interval; d = Cohen’s D; p = level of significance. * significant time effect.

**Supplementary table 6.** Detailed results of the post-hoc with Bonferroni corrections for significant time x condition interaction for relative changes.

|  | | Δ_POST_ vs. Δ_P07_ | | | Δ_POST_ vs. Δ_P14_ | | | Δ_P07_ vs. Δ_P14_ | | |
| --- | --- | --- | --- | --- | --- | --- | --- | --- | --- | --- |
| Variable | Condition | Mean difference  (95% CI) | d | p | Mean difference  (95% CI) | d | p | Mean difference  (95% CI) | d | p |
| PTT | CON | -3.992 (-14.386; 6.402) | -0.261 | 1.000 | -3.759 (-14.153; 6.635) | -0.246 | 1.000 | 0.233 (-10.161; 10.627) | -0.015 | 1.000 |
|  | SLOW_DS_ | 9.421 (-0.973; 19.815) | 0.616 | 0.142 | 11.716 (1.322; 22.110) | 0.767 | 0.011* | 2.296 (-8.099; 12.690) | 0.150 | 1.000 |
|  | MOD_DS_ | 10.674 (0.280; 21.068) | 0.699 | 0.037* | 16.661 (6.267; 27.055) | 0.069 | 0.001* | 5.987 (-4.407; 16.381) | -0.392 | 1.000 |
|  | FAST_DS_ | 12.003 (1.609; 22.397) | 0.785 | 0.008* | 14.238 (3.844; 24.632) | -0.932 | 0.001* | 2.235 (-8.159; 12.629) | -0.379 | 1.000 |

PTT = Peak Twitch Torque; CON = Control Session; SLOW_DS_ = Dynamic stretching performed at a slow velocity; MOD_DS_ = Dynamic stretching performed at a moderate velocity; FAST_DS_ = Dynamic stretching performed at a fast velocity; PRE = Pre-test; POST = test performed immediately after experimental condition; P07 = test performed 7 minutes after experimental condition; P14 = test performed 14 minutes after experimental condition; CI = Confidence Interval; d = Cohen’s D; p = level of significance. *significant difference from CON.
